# Human adipose tissue-derived mesenchymal stem cells and their extracellular vesicles modulate lipopolysaccharide activated human microglia

**DOI:** 10.1038/s41420-021-00471-7

**Published:** 2021-05-10

**Authors:** Marta Garcia-Contreras, Avnesh S. Thakor

**Affiliations:** Interventional Regenerative Medicine and Imaging Laboratory, Department of Radiology, Stanford University, Palo Alto, CA 94304 USA

**Keywords:** Mesenchymal stem cells, Molecular neuroscience

## Abstract

Neurodegenerative diseases (NDs), such as Alzheimer’s disease (AD), are driven by neuroinflammation triggered by activated microglial cells; hence, the phenotypic regulation of these cells is an appealing target for intervention. Human adipose tissue-derived mesenchymal stem cells (hAD-MSCs) may be a potential therapeutic candidate to treat NDs given their immunomodulatory properties. Evidence suggests that the mechanism of action of hAD-MSCs is through their secretome, which includes secreted factors such as cytokines, chemokines, or growth factors as well as extracellular vesicles (EVs). Recently, EVs have emerged as important mediators in cell communication given, they can transfer proteins, lipids, and RNA species (i.e., miRNA, mRNA, and tRNAs) to modulate recipient cells. However, the therapeutic potential of hAD-MSCs and their secreted EVs has not been fully elucidated with respect to human microglia. In this study, we determined the therapeutic potential of different hAD-MSCs doses (200,000, 100,000, and 50,000 cells) or their secreted EVs (50, 20, or 10 µg/ml), on human microglial cells (HMC3) that were activated by lipopolysaccharides (LPS). Upregulation of inducible nitric oxide synthase (iNOS), an activation marker of HMC3 cells, was prevented when they were cocultured with hAD-MSCs and EVs. Moreover, hAD-MSCs inhibited the secretion of proinflammatory factors, such as IL-6, IL-8, and MCP-1, while their secreted EVs promoted the expression of anti-inflammatory mediators such as IL-10 or TIMP-1 in activated microglia. The present data therefore support a role for hAD-MSCs and their secreted EVs, as potential therapeutic candidates for the treatment of NDs.

## Introduction

Microglia are the resident macrophages of the central nervous system (CNS) and participate in CNS homeostasis^1,2^. In response to injury, microglia change their state/polarization from a classic M1 phenotype to an activated M2 phenotype; while the M1 phenotype is toxic to neurons by secreting proinflammatory cytokines and reactive oxygen and nitrogen species, the M2 phenotype secretes anti-inflammatory cytokines, has enhanced phagocytic activity, and releases neurotrophic factors^2^. Hence, persistent activation of M1 microglia has been associated with the initiation and progression of neurodegenerative diseases (NDs), such as Alzheimer’s disease (AD)^3,4^. Recent genome-wide association studies have found that AD risk loci are in, or near, genes that are preferentially or uniquely expressed in microglia^5^. Taken together, these data strongly support the involvement of microglia in the early steps of AD thereby making them an important potential therapeutic target. Indeed, inhibiting proinflammatory M1 microglia and facilitating their switch to a protective and anti-inflammatory M2 phenotype may prove to be an important therapeutic strategy for treating neuroinflammatory-related diseases such as AD.

Mesenchymal stem cells (MSCs) are multipotent stem cells that have been shown to exert immunomodulatory properties^6–11^, including immunosuppression of T lymphocytes and dendritic cells^12^, inhibition of B-cell proliferation^13^, and immunomodulation of other immune cells such as natural killer cells^14^. Human adipose tissue-derived MSCs (hAD-MSCs) are particularly promising for clinical therapy given they can be easily and repeatable harvested from patients using minimally invasive techniques^15^. In animal models and clinical trials associated with AD, hAD-MSCs have shown to ameliorate AD disease symptoms by modulating inflammatory mediators as well as microglia proliferation, polarization, and phagocytic activity^16–18^. The mechanism underlying this effect has been shown, in part, to be due to the paracrine activity of MSCs via their secretome, which includes their release of soluble cytokines, growth factors, and extracellular vesicles (EVs)^19,20^. EVs are secreted small (~30–200 nm in diameter) lipid vesicles that have emerged as important mediators in intercellular communication by transferring RNA, DNA, proteins, and lipids between cells^21,22^. Furthermore, MSC-secreted EVs have been shown to have similar therapeutic properties when compared to their parent MSCs and recent studies have shown that EVs can also polarize microglia from an M1 to an M2 phenotype^23^. Given that EVs have a low immunogenicity, they therefore hold great potential as a potential cell-free therapy^24,25^.

To our knowledge, few studies have investigated the underlying mechanism of hAD-MSCs and their secreted EVs, in human microglia by focusing on their immunomodulatory phenotype. Given that the genomic responses in mouse models of inflammation do not correlate well with human genomic changes^26^ (i.e., molecular pathways and signaling functions differ between rodent and human microglia^1^), we investigated the effect of hAD-MSCs and their EVs, in a human microglia cell line (human microglia clone 3 (HMC3) cells)^27,28^.

## Results

### The effect of lipopolysaccharides (LPS) on HMC3 cells

**Fig. 1:**
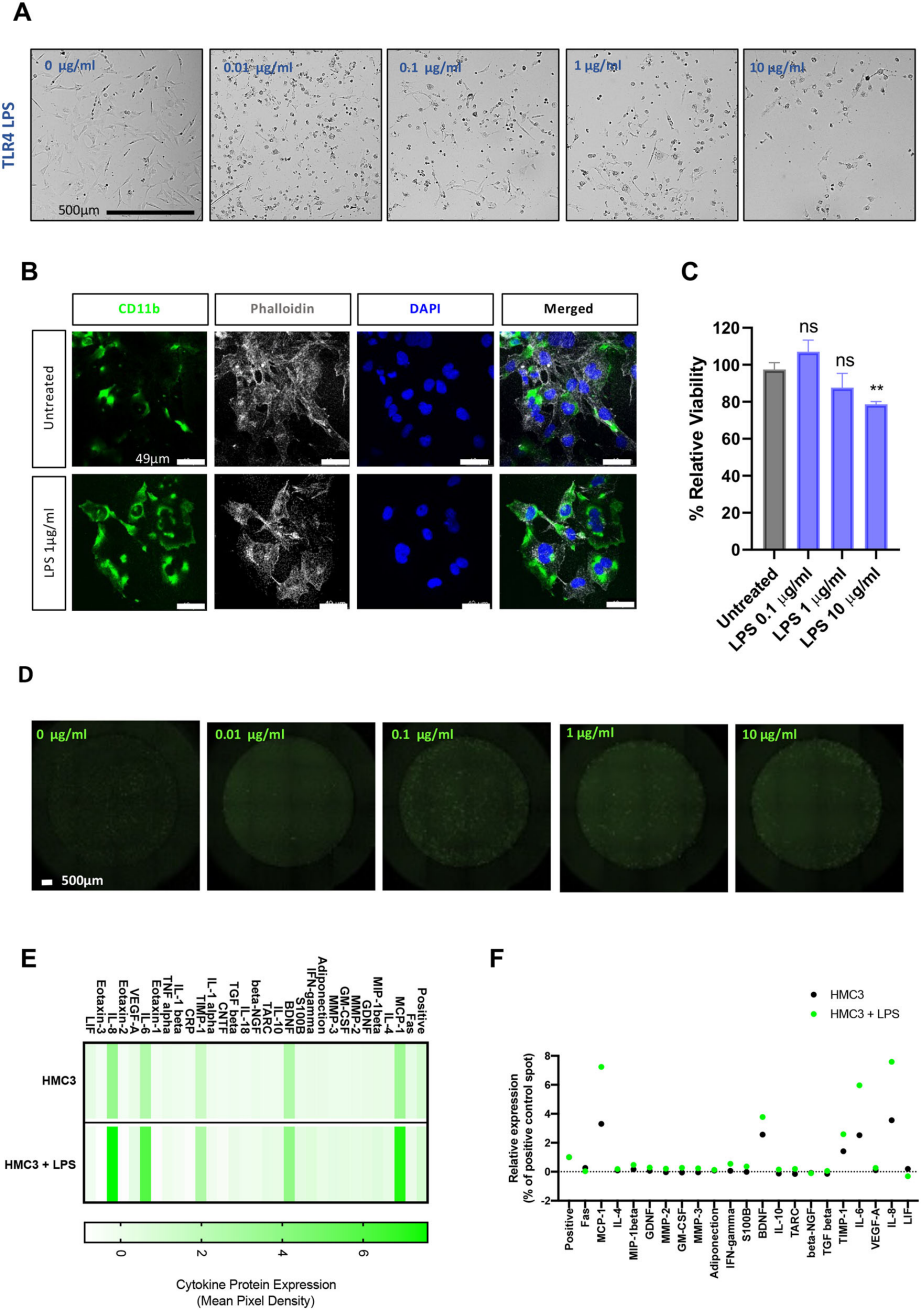
Effects of LPS on HMC3 microglia. **A** Representative phase-contrast images show morphological changes in HMC3 microglia after 24 h incubation with different doses of LPS. Scale bar, 500 μm. **B** Immunofluorescence staining for CD11b (green fluorescent signal), phalloidin (white), and nuclear DAPI staining (blue) was performed. Scale bar, 49 μm. **C** Cell viability after 24 h incubation with different doses of LPS was determined by the XTT assay. Values for each treatment are expressed as percentages of the corresponding control (cells untreated). The results shown are the mean ± SEM of triplicates. Double asterisks (**) indicate a significant difference from the LPS-activated group vs control (*p* < 0.01). **D** LPS-induced ROS generation in HMC3 microglia cells in response to 24 h LPS exposure was determine using Celigo imaging cytometer. Representative images are shown. **E** Heat map showing relative cytokines expression. Collected supernatants were subject to cytokine array blots containing 30 different human neurologically relevant proteins. **F** Representation of relative cytokines expression. The values are shown as mean pixel density.

To assess the effect of different magnitudes of toll-like receptor 4 (TLR4) activation, HMC3 cells were incubated with increasing concentrations (0.01–10 µg/ml) of LPS for 24 h. At low concentrations of LPS, HMC3 cells had an ameboid spherical morphology, characteristic of activated microglia (Fig. 1A, B). Furthermore, LPS increased the expression of CD11b (Fig. 1B), a beta-integrin marker of microglia. While at low concentrations (0.1–1 µg/ml) of LPS there was microglia activation, higher concentrations (10 µg/ml) caused cell death (*p* < 0.01) (Fig. 1B). Our data show that LPS-induced intracellular reactive oxygen species (ROS) accumulation even at lower concentrations ≤1 µg/ml (Fig. 1D). To further understand the effect of inflammation on HMC3 cells, the relative expression of cytokines and chemokines in the conditioned media from 1 µg/ml LPS-treated and control cells was measured (Fig. 1E, F); here, monocyte chemoattractant protein-1 (MCP-1), brain-derived neurotrophic factor (BDNF), TIMP metallopeptidase inhibitor 1 (TIMP-1), interleukin 6 (IL-6), and IL-8 were all increased upon LPS activation.

### Characterization of hAD-MSCs and their secreted EVs

**Fig. 2:**
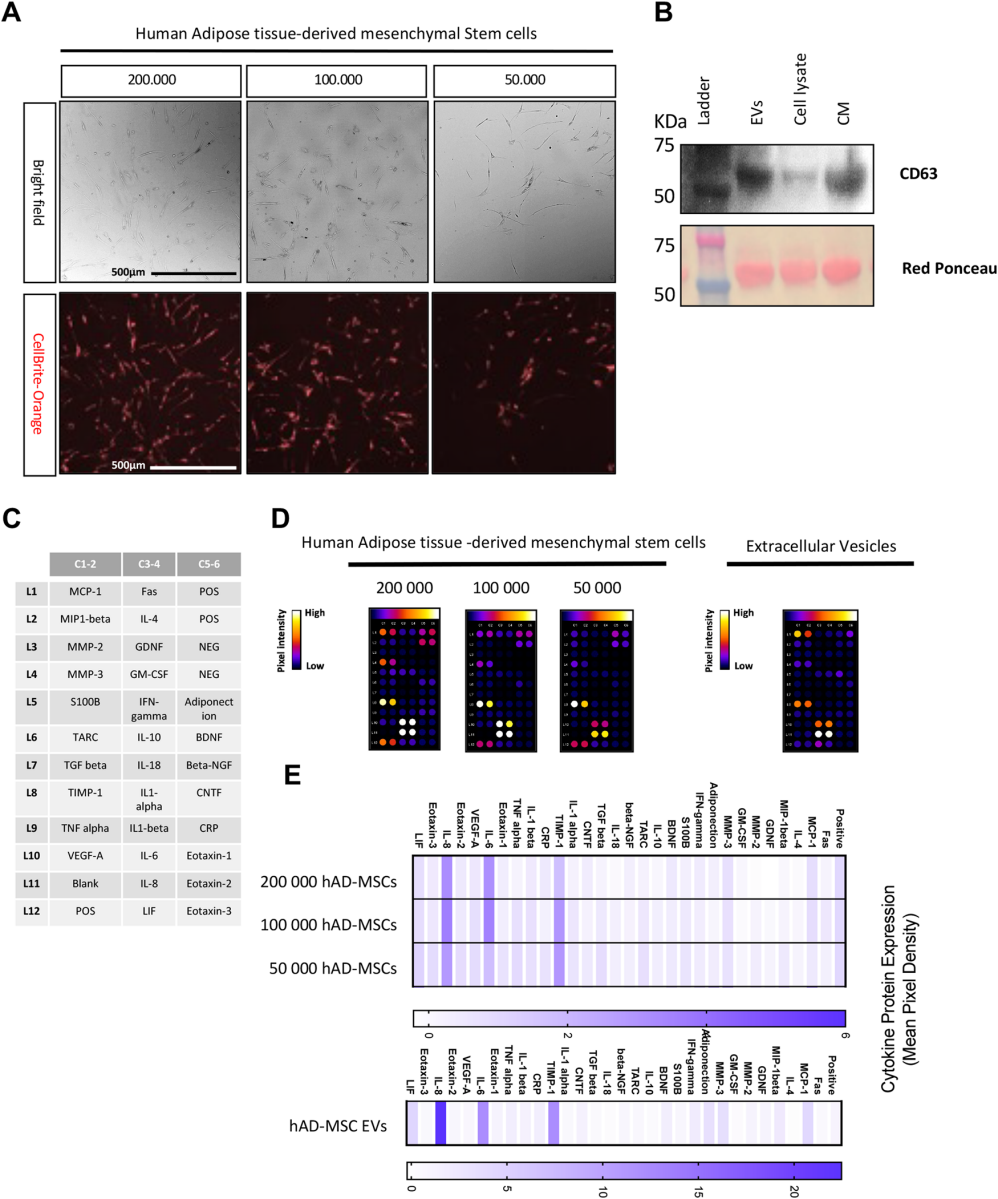
Human adipose tissue-derived stem cells and extracellular vesicles characterization. **A** Morphology of isolated hAD-MSCs after 24 h in culture. Representative images are shown. **B** Western blotting was used to confirm the abundances of extracellular vesicle marker CD63 in isolated extracellular vesicles (EVs), cell culture supernatants (CM), and cell lysates. Ponceau red staining is representative of total protein content. **C** Gene map of cytokine array containing 30 different human neurologically relevant proteins; all spots are in duplicate and **D** cytokine array membranes. **E** Heat map showing relative cytokines expression. The values are shown as the mean pixel density.

hAD-MSCs were isolated using Lipogems^®^ technology which is a nonenzymatic method for AD-MSC isolation as previously described^29^. Three different concentrations of hAD-MSCs (200,000, 100,000, and 50,000 cells) were cultured and their morphological features assessed after 24 h by phase-contrast microscopy (Fig. 2A). Representative images show that hAD-MSCs had the typical MSC spindle and multipolar shape morphology. To determine the role of secreted EVs by hAD-MSCs, we collected the conditioned media and EVs. Expression of EV markers in the fractionated vesicles and conditioned medium were also evaluated (Fig. 2B). Western blot analysis demonstrated enrichment of CD63 (i.e., a classical EV marker) in both isolated EVs and the CM when compared to cell lysates. Ponceau red staining show total protein content.

Analysis of the secretome from hAD-MSCs (Fig. 2C–E) showed an increased expression of TIMP-1, IL-6, and IL-8; indeed, these cytokines were highly expressed in EVs when compared to parental MSCs (Fig. 2D, E).

### Coculture of HMC3 cells with hAD-MSC or their secreted EVs

**Fig. 3:**
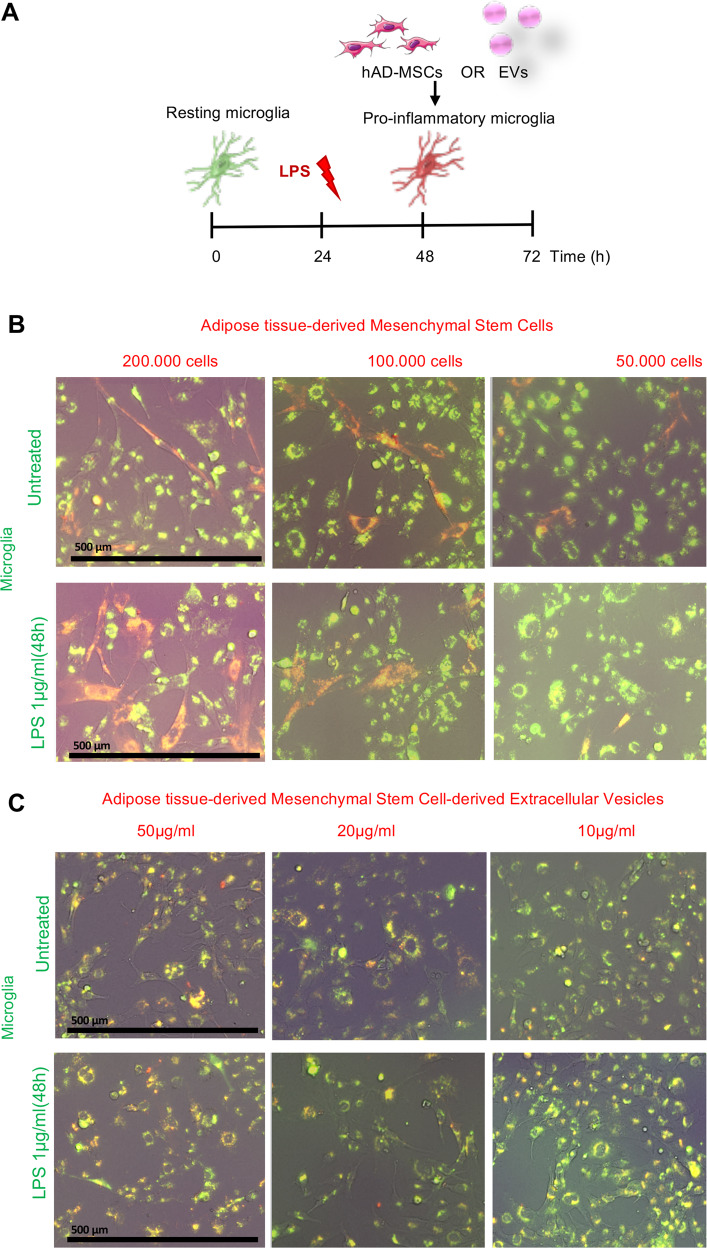
Coculture of human HMC3 microglia cells and human adipose tissue-derived mesenchymal stem cells OR extracellular vesicles. **A** Schematic overview of the coculture protocol. **B** Fluorescence images of culture HMC3 microglia cells (green) and human adipose tissue-derived mesenchymal stem cells (red) in the presence or absence of 1 µg/ml LPS. **C** Fluorescence images of culture HMC3 microglia cells (green) and human adipose tissue-derived mesenchymal stem cell extracellular vesicles (red) in the presence or absence of 1 µg/ml LPS. Scale bar 500 µm.

Untreated and activated (1 µg/ml LPS) HMC3 cells were cocultured with different ratios of hAD-MSCs and EVs for 24 h (Fig. 3A). When untreated HMC3 cells were cocultured with hAD-MSCs, they showed a typical resting morphology of elongated and ramified microglia, similar to our control experiment (Fig. 1A). While the addition of LPS (1 µg/ml) resulted in HMC3 cells forming an ameboid spherical morphology, coculturing with hAD-MSCs at 200,000 and 100,000 cells (Fig. 3B) or EVs at 20 and 10 µg/ml (Fig. 3C) showed a significant decrease in the amount of ameboid cells. In addition, images from the Celigo image cytometer revealed colocalization (yellow) of CellBrite Orange labeled isolated EVs by CellBrite Green labeled HMC3 cells.

### Characterization of activated HMC3 cells following coculture with hAD-MSCs or their secreted EVs

**Fig. 4:**
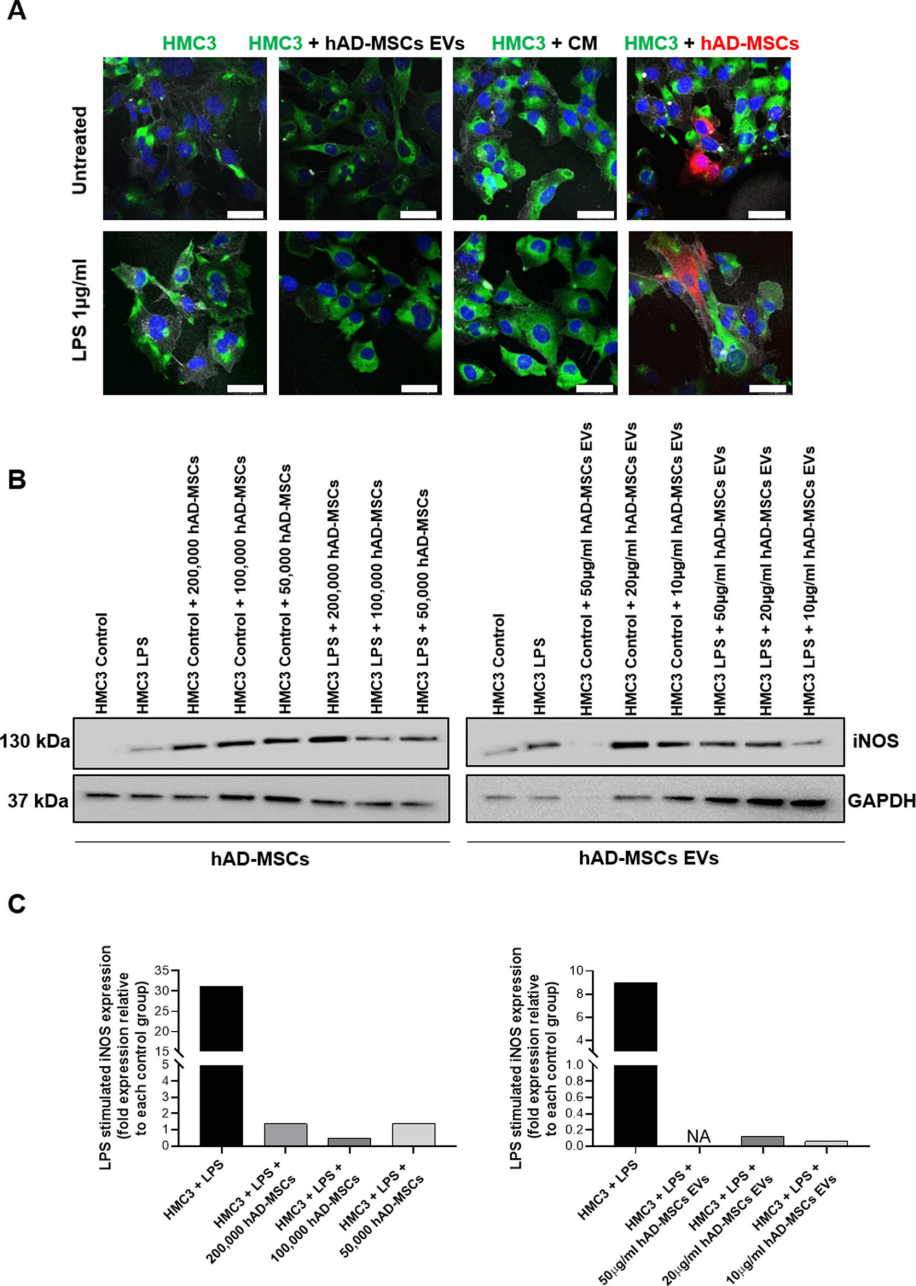
Effect of hAD-MSCs and hAD-MSC EVs on the immunoinflammatory phenotype of microglia. **A** Representative immunofluorescence images with staining for M1 marker CD11b (green), F-actin phalloidin (white), nuclei with DAPI (blue), and hAD-MSCs labeled with CellBrite (red). Scale bar = 49µm. **B** Western blots showing the protein expression of inducible nitric oxide (iNOS) and GAPDH in control and stimulated (with LPS) conditions when HMC3 cells were co-cultured alone or with hAD-MSCs or hAD-MSCs EVs. **C** Quantification of the Western Blot bands shown above. NA indicates a not applicable value due to limited signal detection for GAPDH.

To investigate the effects on microglia phenotype polarization, HMC3 cells were activated with 1 µg/ml LPS for 24 h and then cocultured with hAD-MSCs or their secreted EVs. We performed an immunofluorescence staining for CD11b, a marker of beta-integrin on microglia and phalloidin, which detects F-actin (Fig. 4A). As previously shown, LPS-treatment upregulated CD11b expression and ameboid morphology in comparison to untreated HMC3 cells. However, when activated microglia were cocultured with hAD-MSCs or their EVs, there was a decrease in CD11b expression and ameboid morphology which was visible after 24 h (Fig. 4A).

**Fig. 5:**
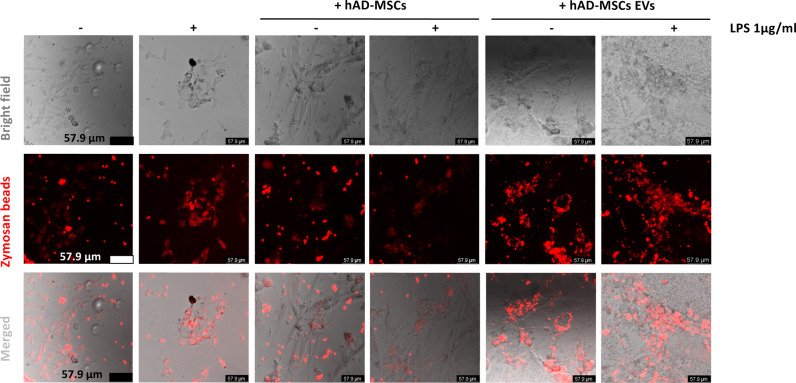
Human adipose tissue-derived stem cells secreted extracellular vesicles enhanced phagocytosis in human HMC3 microglia. Representative images of untreated or LPS-stimulated HMC3 microglia capacity of phagocytize fluorescent-labeled latex beads.

Following LPS activation, inducible nitric oxide synthase (iNOS) increased in HMC3 cells (Fig. 4B, C). On the contrary, in the presence of hAD-MSCs and hAD-MSC EVs, the level of iNOS was decreased (Fig. 4C). Furthermore, we observed that labeled zymosan particles were phagocytosed by HMC3 cells treated with LPS (Fig. 5). However, this effect was inhibited when HMC3 cells were cocultured with hAD-MSCs, but not when they were cocultured with hAD-MSCs EVs.

### Cytokine secretion by activated HMC3 cells

**Fig. 6:**
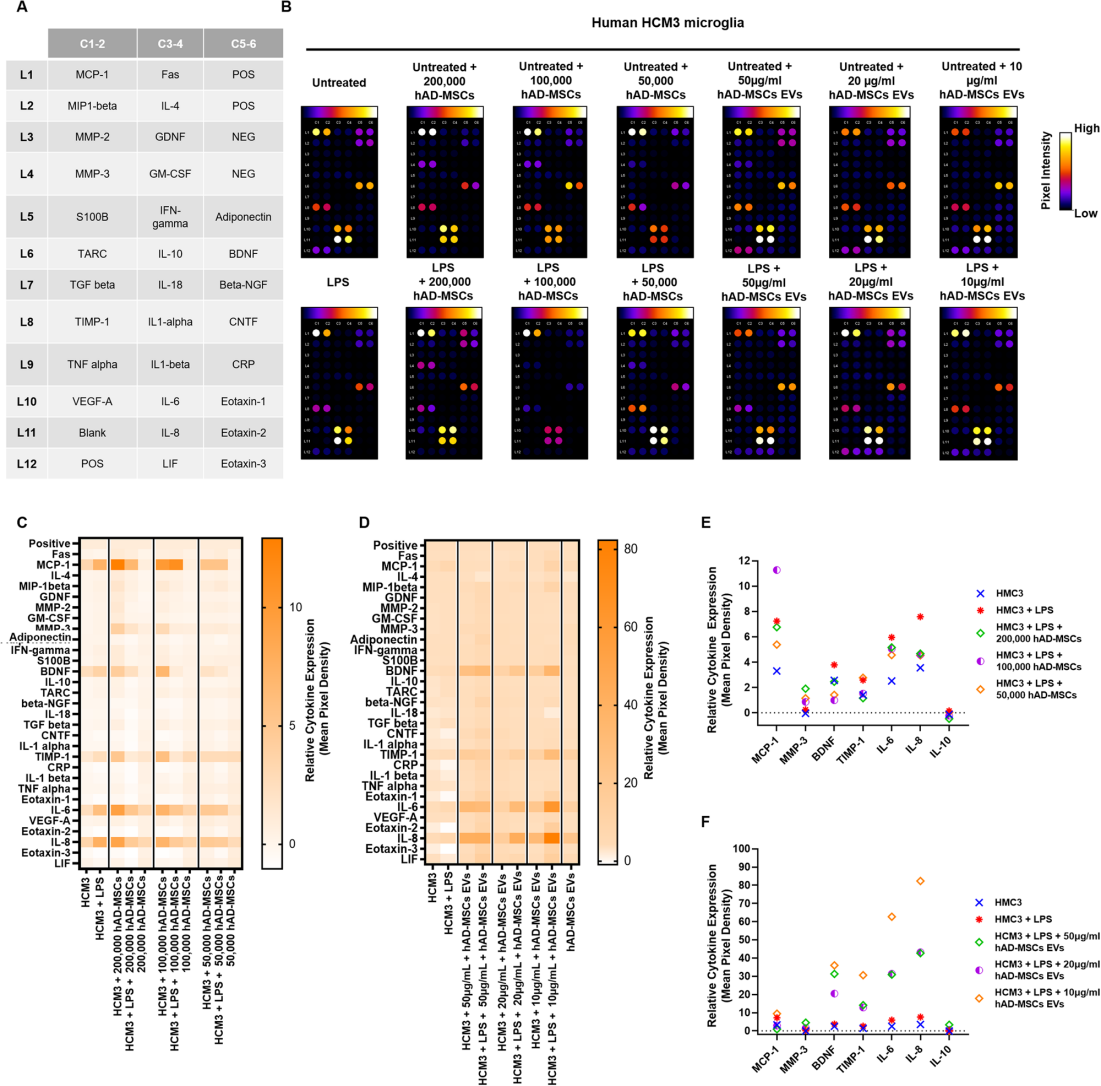
Changes in cytokine production in untreated or LPS-stimulated HMC3 microglia mediated by hAD-MSCs and hAD-MSC EVs. **A** Gene map of cytokine array. **B** Cytokine arrays, in which human HMC3 microglia have been cultured with human adipose tissue-derived stem cells or extracellular vesicles; all spots are in duplicate. **C**, **D** Heat map showing relative cytokine expression. **E**, **F** Relative expression of select cytokines; values are shown as the relative mean pixel density of the duplicate spots.

Cytokine secretion in HMC3 activated or nonactivated microglia in the presence or absence of hAD-MSCs or EVs was determined (Fig. 6A, B). Following LPS activation of HMC3 cells, there were increases in IL-6 and IL-8 when compared to resting untreated microglia (Fig. 6C–F). Treatment with hAD-MSCs attenuated the secretion of IL-8, as well as IL-6 to a lesser degree, from activated microglia (Fig. 6C, E and Supplementary Fig. 1). In contrast, the secretion of IL-8 or IL-6 was upregulated in the presence of hAD-MSC EVs (Fig. 6D, F). Similarly, activation of HMC3 cells resulted in a twofold increase in MCP-1 which was decreased by both hAD-MSCs (50,000 cells) and hAD-MSCs EVs (50 and 20 µg/ml), with the effect being greater for EVs (Fig. 6D, F and Supplementary Fig. 1). In addition, in activated microglia, the protein level of TIMP-1 was also significantly upregulated by EVs and downregulated by high hAD-MSCs doses (200,000 and 100,000 cells), while IL-10 levels were upregulated by higher doses of hAD-MSCs EVs (50 and 20 µg/ml). BDNF protein expression was also upregulated by EVs and downregulated by all hAD-MSCs doses (Fig. 6E, F and Supplementary Fig. 2).

## Discussion

Although previous studies have examined the neuroimmunomodulatory potential of MSCs and their secretome, in mouse microglia^30,31^, this is the first report demonstrating the therapeutic efficacy of hAD-MSCs and their EVs, on human microglia. Here, we show that hAD-MSCs and their secreted EVs can modulate the phenotype of activated human microglia.

The therapeutic and regenerative potential of MSCs and their secretome have attracted much attention in recent years, especially for the treatment of neuroinflammatory and NDs^32–34^. MSCs are multipotent stromal cells which have been shown to have immunomodulatory properties, low tumorigenic risk, and are able to efficiently home to areas of injury^32,35,36^. Many studies have reported that MSCs exhibit their immunomodulatory function via EVs, which are secreted into their surrounding microenvironment^37–39^. The use of MSC-secreted EVs could provide several advantages over parental MSCs, given they are less likely to cause pulmonary embolism following intravenous injection, have lower immunogenicity, and have been shown to cross the blood brain barrier^25,40,41^. Hence, in the present study, we tested both hAD-MSCs and their secreted EVs, on human microglia.

In order to activate HMC3 cells, we used the bacterial endotoxin LPS, which is the most commonly used ligand of TLR4^28,42^. TLRs are a class of pattern-recognition receptors in the innate immune system, and TLR4 is a key regulator of inflammation that has been shown to play an essential role in NDs such as AD^43,44^. LPS was shown to increase ROS production in microglia and activate them to a classical “M1” proinflammatory phenotype; this was supported by their morphological change from a ramified to an ameboid shape with increased CD11b expression. In addition, LPS-activated microglia demonstrated an increase in expression of iNOS^45^, which has been associated with neuronal cell death and neurodegenerative conditions^46^, as well as cytokines including MCP-1, IL-6, and IL-8, which have been shown to be present in chronic inflammatory diseases^47–51^.

Treating activated microglia with hAD-MSCs, or their secreted EVs, resulted in a decrease in CD11b expression and ameboid morphology as well as a decrease in expression of iNOS and MCP-1^31^. It has been previously reported that nitric oxide signaling is involved in brain memory development through nNOS and neuroinflammation through iNOS which has also been shown to mediate several CNS inflammatory processes^52^. In relation to CNS diseases such as AD, it has been reported that nNOS and iNOS contribute to the progression of AD, with nNOS having a stronger effect^53^. Removal of iNOS in transgenic AD mice, or the use of iNOS inhibitors such as l-NAME (i.e., a general NOS inhibitor) or 1400 W (i.e., a specific iNOS inhibitor), results in improved behavioral dysfunction and protection against Aβ neurotoxicity in models of AD^53,54^. Furthermore, the use of nNOS inhibitors has shown to be beneficial in other models of CNS diseases such as Parkinson’s disease and ALS.

While hAD-MSCs decreased the expression of IL-6 and IL-8, their secreted EVs increased the level of these cytokines; this discrepancy can be partially explained by the fact that IL-6 and IL-8 are highly expressed in EVs and hence any increase in these levels might come from EVs rather than the microglia. Interestingly, hAD-MSCs only were also able to decrease the phagocytic capacity of activated microglia. Overall, our data suggest that hAD-MSCs and their secreted EVs are able to inhibit the proinflammatory effects of human microglia by inhibiting M1 polarization and promoting instead their M2 polarization.

Our results also show that hAD-MSCs increased the secretion of factors, such as TIMP-1, which is known to be a regulator of hAD-MSCs function and proliferation^55,56^ and leukemia inhibitory factor which maintains the stem state of MSCs^57^; both these factors were higher with a lower number of hAD-MSCs (i.e., 50,000 cells). In contrast, while IL-6, which mediates several therapeutic effects of hAD-MSCs^58^, increased in proportion to the number of hAD-MSCs, IL-8 did not change with the number of hAD-MSCs. Taken together, these results show that the number of hAD-MSCs is important when considering their therapeutic effects which will be important for their “dosing” as they are clinically translated.

Both IL-10 and TIMP-1 are important anti-inflammatory mediators which act as negative-feedback regulators to maintain a balanced immune response. Indeed, IL-10 plays a crucial anti-inflammatory role in microglia^59^ and has been shown to be involved in the cognitive decline of Alzheimer patients, where slow decliners have higher levels of IL-10^60^. In our study, hAD-MSC EVs (50 µg/ml) increased IL-10 levels which may represent an important neuroprotective effect. TIMP-1 is expressed by variety of cell types including astrocytes^61^, and is known to be able to shift microglia from an M1 to an M2 state^62,63^. In LPS-activated microglia cocultured with EVs, TIMP-1 was increased supporting an anti-inflammatory role of EVs on microglia. BDNF, which is a neurotrophin that is required for the survival of specific neuronal populations given its ability to facilitate axonal and dendritic growth and synaptogenesis^64,65^, has also been shown to be associated with cognitive decline in AD patients, with levels decreasing in advanced stages^66,67^. Interestingly, EVs were able to increase the levels of BDNF in LPS-activated microglia which, in turn, may be beneficial for them to ameliorate neural dysfunction.

In conclusion, we demonstrate that hAD-MSCs and their secreted EVs can prevent a proinflammatory microglia phenotype. These findings support a role for hAD-MSC EVs as a potential cell-free therapy for the treatment of neuroinflammatory diseases including AD. Further studies are required to determine the content of hAD-MSC-secreted EVs and the different molecular mechanisms of their action to understand how they exactly modulate microglia both in cell culture as well as in animal models.

## Materials and methods

### Cell lines and culture

HMC3 cells were purchased from ATCC (ATCC^®^ CRL-3304™) and grown in EMEM Media (ATCC, VA, USA), supplemented with 10% fetal bovine serum (Gibco) and 1% penicillin/streptomycin (Gibco-Life Technologies, USA) in multilayer flasks (Nest Scientific, USA) at 37 °C and 5% CO_2_ in a forced air incubator. hAD-MSCs were kindly donated by Dr. Ricordi’s Lab (Diabetes Research Institute, University of Miami, Miami, FL, USA); details of their isolation and characterization have been previously described^68,69^. These cells were grown in Prime-XV expansion serum-free Media (91135, Fujifilm Irvine Scientific, CA, USA) and 1% penicillin/streptomycin (Gibco-Life Technologies, USA) at 37 °C and 5% CO_2_ in a forced air incubator. For all experiments, hAD-MSCs were used up to passage number 5.

### Collection and characterization of hAD-MSC-secreted EVs

hAD-MSCs were grown to 80–90% confluence, and then maintained in serum-free Prime-XV media (91135, Fujifilm Irvine Scientific, CA, USA) which was collected after 24 h and then centrifuged at 500 × *g* for 5 min followed by 2000 × *g* for 30 min at 4 °C to remove cell debris and large apoptotic bodies. The media were then filtered through a 0.45 µm filter and stored. Next, a polyethylene glycol (PEG) solution was prepared to precipitate EVs. PEG with a molecular weight of 4000 (25322-68-3, Bioworld, OH, USA) was combined with filtered water and sodium chloride (1 M) to make a twofold concentrated (2x) stock solution. The 2x stock solution was added to an equal volume of serum-free conditioned media. In order to isolate EVs, the collected media were added to an equal volume of a 2x PEG stock solution at 4 °C. After the 2x PEG solution was added, samples were mixed thoroughly by inversion, and incubated at 4 °C overnight. The next day, samples were centrifuged in a tabletop centrifuge at 3000 × *g* for 1 h at 4 °C. Conical tubes were then decanted, and allowed to drain for 5 min, tapping occasionally to remove excess PEG. The resulting pellet was suspended in 100 μl of particle-free PBS (pH 7.4; Gibco-Life Technologies, USA) and the samples finally stored at −80 °C. Prior to their use, the protein content in EVs was quantified using a BCA protein assay following the manufacturer’s recommendations (Pierce, Thermo Scientific) after samples were lysed with RIPA buffer (89900, Pierce, Thermo Scientific) which was supplemented with a protease inhibitor mixture (11697498001, Sigma-Aldrich).

### Fluorescence labeling of HMC3 cells, hAD-MSCs, and their secreted EVs

To enable discrimination between the different cell types and visualization of EVs, hAD-MSCs and their secreted EVs were stained with CellBrite Orange (30022, Biotium, CA, USA) and HMC3 cells were stained with CellBrite Green (30021, Biotium, CA, USA). In brief, all cells were detached from culture flasks by trypsinization and then quantified using a Countess II Automated Cell Counter (Invitrogen, Thermo Fisher) before being suspended at a density of 1 × 10^6^ cells/ml in cell culture media. For cell staining, 5 µl of the CellBrite labeling solution was added per 1 ml of cell suspension for 20 min at 37 °C. The mixture was then centrifuged at 350 × *g* for 5 min to create a cell pellet and to enable the staining solution to be removed. The cells were then washed with PBS and placed in fresh media. For EVs, the pellets were resuspended in 100 µl PBS (1x), mixed with 10 μl Orange CellBrite solution, and incubated for 30 min at 37 °C. The labeling reaction was stop by adding 100 µl of PEG (2x) stock solution and the EVs retrieved by centrifugation at 5000 × *g* for 30 min.

### Coculture experiments

Microglia can be activated via TLRs, a class of pattern-recognition receptors in the innate immune system. Microglial TLR 4 is a key regulator of inflammation that may play a role in AD^43^. Bacterial endotoxin LPS is the most commonly used ligand of TLR4 which can induce microglial activation. To asses if hAD-MSCs and their secreted EVs can modulate microglial activation in response to LPS, HMC3 cells were plated at 200,000 cells per well in six-well plates or chamber slides and incubated for 24 h in the presence of 1 µg/ml LPS (Sigma-Aldrich), with or without, hAD-MSCs or EVs. To determine if there was any dose-dependent effects, HMC3 cells were kept consistent at 200,000 cells (with or without LPS stimulation) and the following groups tested: hAD-MSCs = 200,000, 100,000, and 50,000 cells and EVs = 50, 20, and 10 µg/ml. Cocultures were live-imaged and analyzed using an Celigo image cytometer (Nexcelom, Lawrence, MA).

### XTT cell proliferation assay

HMC3 cells were seeded in 96-well tissue culture plates containing complete growth medium EMEM (ATCC, VA, USA), supplemented with 10% fetal bovine serum (Gibco) and 1% penicillin/streptomycin (Gibco-Life Technologies, USA) and were cultured in the presence or absence of different doses of LPS (0.1–10 µg/ml). After treatments, cell viability was assessed using a XTT cell proliferation Cayman chemical assay kit (no. 10010200; Cayman Chemical). XTT reagent (10 µl) was added to each well, which was subsequently incubated for 2 h at 37 °C. The culture medium was discarded, and 100 µl of the crystal dissolving solution was added to dissolve the formazan dye. Then, absorbance was measured at 570 nm using a microplate spectrophotometer system (Tecan Infinite M200 plate reader). The obtained data were normalized to the absorbance value, measured obtained at 570 nm from nontreated cells (100%), and expressed as a percentage of the control ± standard error of the mean (SEM).

### ROS assay

ROS were measured using 2′,7′-dichlorodihydrofluorescein diacetate (DCFH-DA) (D6883-50MG, sigma). DCFH-DA is a cell-permeable nonfluorescent probe which is hydrolyzed by intracellular esterases, thereby trapping it within the cell. This nonfluorescent molecule can then be oxidized by ROS, which then turns it into fluorescent dichlorofluorescin (DCF). The level of intracellular fluorescence is therefore proportional to the amount of intracellular ROS production with a linear dynamic range. All experiments were performed in 96-well plates. HMC3 were incubated for 45 min with 10 μM of DCFH-DA, and the fluorescence emission of DCF measured at 488 nm using a Celigo image cytometer (Nexcelom; Lawrence, MA).

### Phagocytosis assay

To quantify the phagocytic capacity of HMC3, cells were grown in glass bottom chamber slides. After treatment, cells were incubated with 5 µl of prelabeled zymosan particles (ab234054, Abcam) for 2 h and then washed by adding cold phagocytosis assay buffer. Cells were analyzed by confocal fluorescent microscopy (SP8 Leica microscope).

### Immunofluorescence staining

Immunofluorescence staining was performed to detect the expression of microglia phenotype markers. Following each experiment, HMC3 cells were washed with PBS, fixed with 4% formaldehyde for 30 min at room temperature, and permeabilized with 0.1% Triton X-100/PBS. Nonspecific staining was blocked with 5% BSA in 0.1% Triton X-100/PBS for 1 h at room temperature. Cells were then incubated overnight at 4 °C with primary antibodies (Table 1). The next day, cells were rinsed with PBS, following which they were incubated with secondary antibodies (Table 1) for 1 h at room temperature. The nuclei were counterstained with DAPI for 5 min at room temperature. Stained samples were mounted using Vectashield fluorescence mounting media (Vector Laboratories Inc, Burlingame, CA, USA). Images were acquired with Leica SP8 inverted confocal microscope equipment (Leica Microsystems, IL, USA).

**Table 1:** Antibody list.

Name	Catalog number	Company	Host	Dilution
Anti-CD63	ab59479	Abcam	Mouse	1:1000
Anti-CD11b	NB110-89474	Novusbio	Rabbit	1:1000
Phalloidin-iFluor™ 647 Conjugate	20555	Cayman Chemical Company	N/A	N/A
Anti-iNOS	NBP2-22119SS	Novusbio	Mouse	1:1000
Anti-GAPDH	Sc-69778	Santa Cruz	Mouse	1:1000
Anti-mouse HRP	7076S	Cell Signaling	Horse	1:5000
Anti-rabbit 488	A11008	Thermo Fisher Scientific	Goat	1:500

### Western blot analysis

Samples were lysed with cold RIPA buffer (Pierce, Thermo Scientific) supplemented with a protease inhibitor mixture (Sigma-Aldrich). The protein concentration was determined using a BCA protein assay following the manufacturer’s recommendations (Pierce, Thermo Scientific). For SDS-PAGE, samples were prepared in Laemmli buffer (Bio-Rad, Hercules, CA, USA), and 25 μg of samples were loaded in 10–12% Mini-PROTEAN TGX™ gels (Bio-Rad) and transferred onto a nitrocellulose membrane. Membranes were blocked with 5% blotting-grade nonfat dry milk (Bio-Rad) in Tris-buffered saline (TBS) for 1 h at room temperature and incubated overnight at 4 °C with the indicated primary antibodies (Table 1) diluted in 0.1% TBS-Tween 20 (TBS-T). Membranes were washed with 0.1% TBS-T, incubated with the indicated secondary antibody (Table 1) for 1 h at room temperature, and then washed again with TBS-T. To create chemiluminescent signals, membranes were incubated with a ECL western blot substrate (GE Healthcare Ltd) and visualized on an IVIS Lumina imager, with Living Image Software.

### Cytokine assays

Cytokine assays were performed using a Human Neuro Discovery Array C2 (RayBiotech, Norcross, GA, USA) according to the manufacturer’s instructions. Membranes precoated with cytokine antibodies were blocked with a blocking buffer at room temperature for 30 min; next, equal volumes of supernatants, collected from HMC3 cells treated, with or without 1 µg/ml LPS in the presence or absence of hADSCs or EVs, were added to replace the blocking buffer and incubated overnight at 4 °C. After washing three times with wash buffer 1 and two times with wash buffer 2, each membrane was incubated with biotin-conjugated antibodies for 2 h followed by a HRP-conjugated streptavidin at room temperature for 30 min. Membranes were developed by Detection buffer C and D mixture and the chemiluminescence was visualized by an IVIS Lumina-III In Vivo Imaging System (PerkinElmer). Densitometry analysis of the array was performed using the NIH Image J software.

### Statistical analysis

All data are expressed as means ± SEM. Two-tailed Student’s *t* tests were applied to assess differences. For all assays (three repetitions, regardless of intra-assay triplicates) were evaluated by the corresponding statistical test. *P* values of <0.05 were considered significant.

## Supplementary information

Table, figures and supplementary figures legends

Supplementary Figure 1

Supplementary Figure 2
