# Author Correction: Human adipose tissue-derived mesenchymal stem cells and their extracellular vesicles modulate lipopolysaccharide activated human microglia

**DOI:** 10.1038/s41420-024-02209-7

**Published:** 2024-10-23

**Authors:** Marta Garcia-Contreras, Avnesh S. Thakor

**Affiliations:** Interventional Regenerative Medicine and Imaging Laboratory, Department of Radiology, Stanford University, Palo Alto, CA 94304 USA

Correction to**:** *Cell Death Discovery* (2021) 7:98 10.1038/s41420-021-00471-7, published online 10 May 2021

In the original version of this article, there was an error in the western blot data in Fig 4B and C, which has now been corrected. In addition, the y axis in Fig 6E and F was split, which has now been removed. The corresponding text for these figures have also been corrected. The authors apologize for this inconvenience.

**Amended Figure 4** 
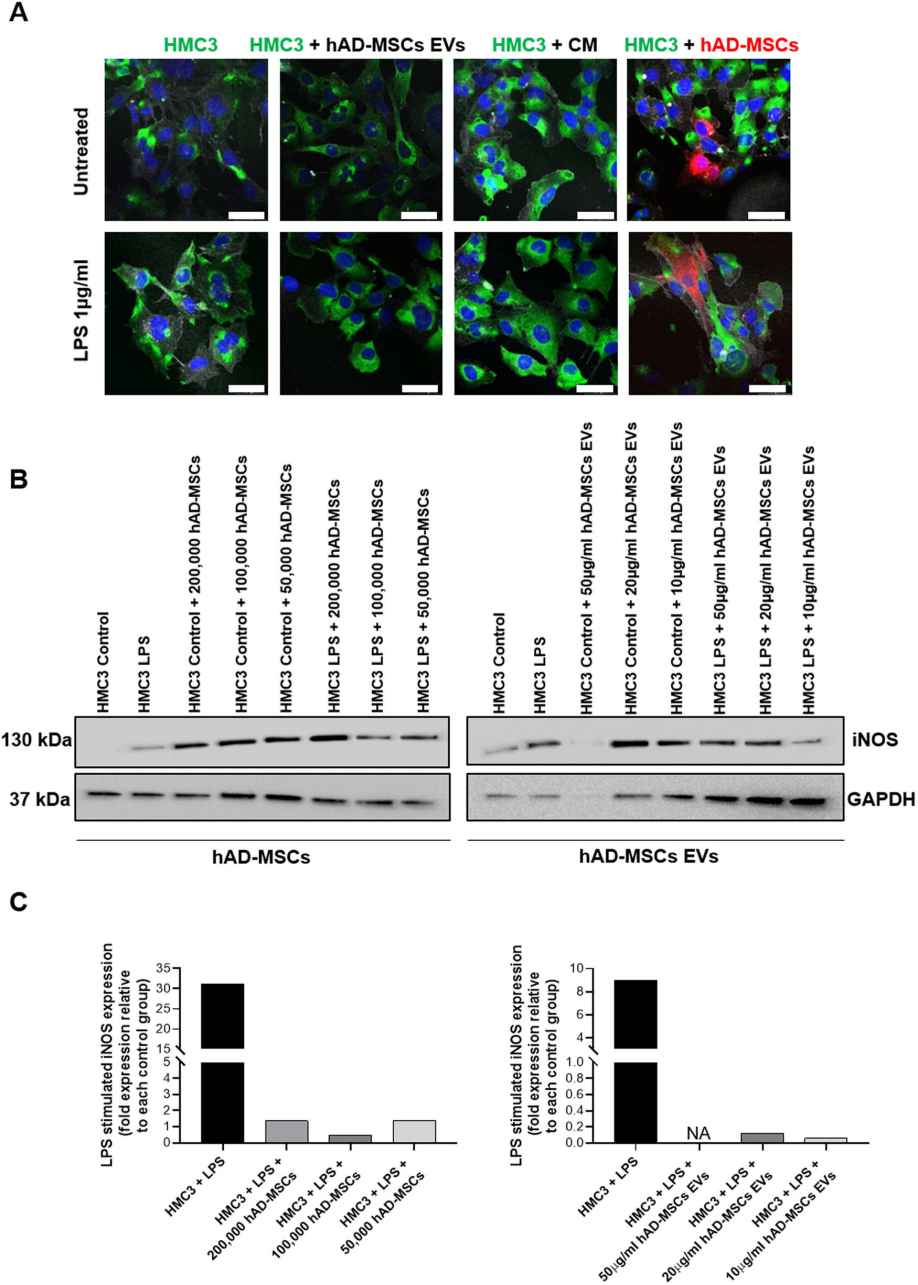


**Original Data - Figure 4** 
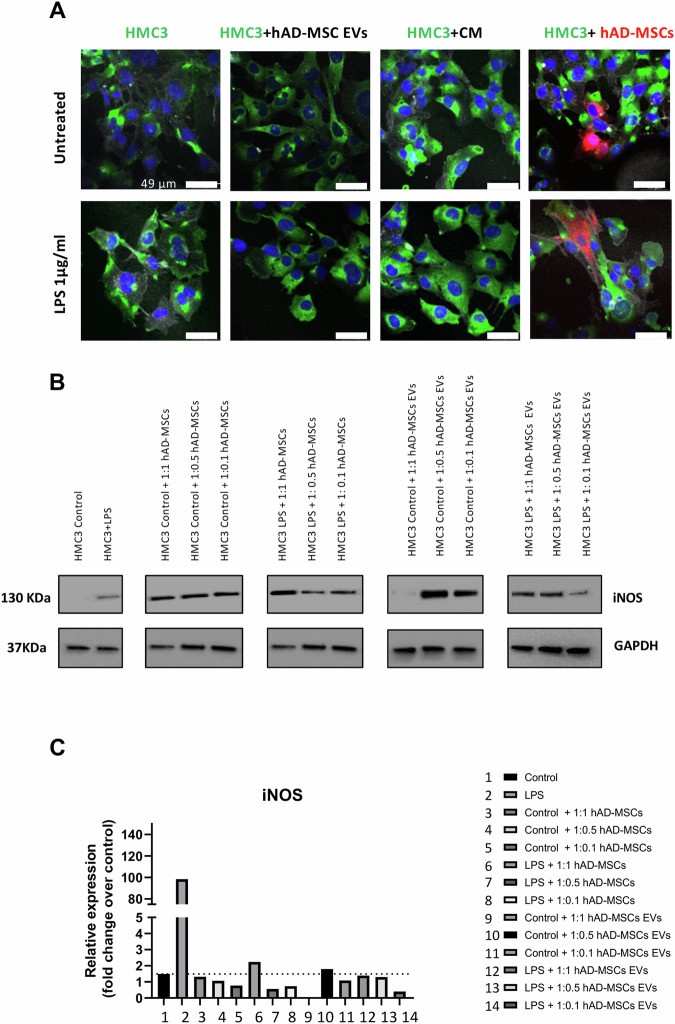


**Legend Corrections:**

**Fig. 4 Effect of hAD-MSCs and hAD-MSC EVs on the immunoinflammatory phenotype of microglia. A** Representative immunofluorescence images with staining for M1 marker CD11b (green), F-actin phalloidin (white), nuclei with DAPI (blue), and hAD-MSCs labeled with CellBrite (red). Scale bar = 49 µm. **B** Western blots showing the protein expression of inducible nitric oxide (iNOS) and GAPDH in control and stimulated (with LPS) conditions when HMC3 cells were co-cultured alone or with hAD-MSCs or hAD-MSCs EVs. **C** Quantification of the Western Blot bands shown above. NA indicates a not applicable value due to limited signal detection for GAPDH.

**Amended Figure 6** 
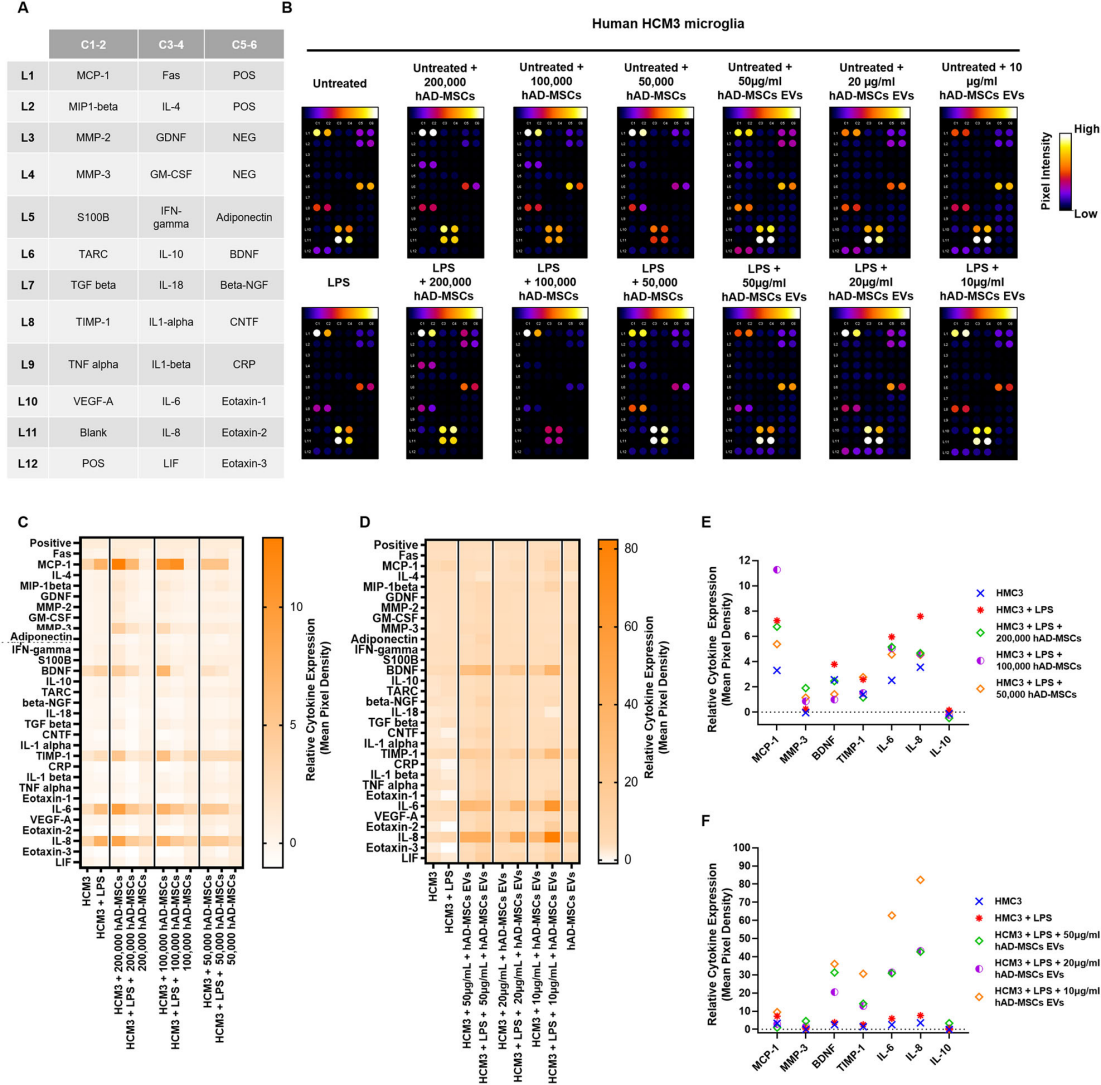


**Original Data - Figure 6** 
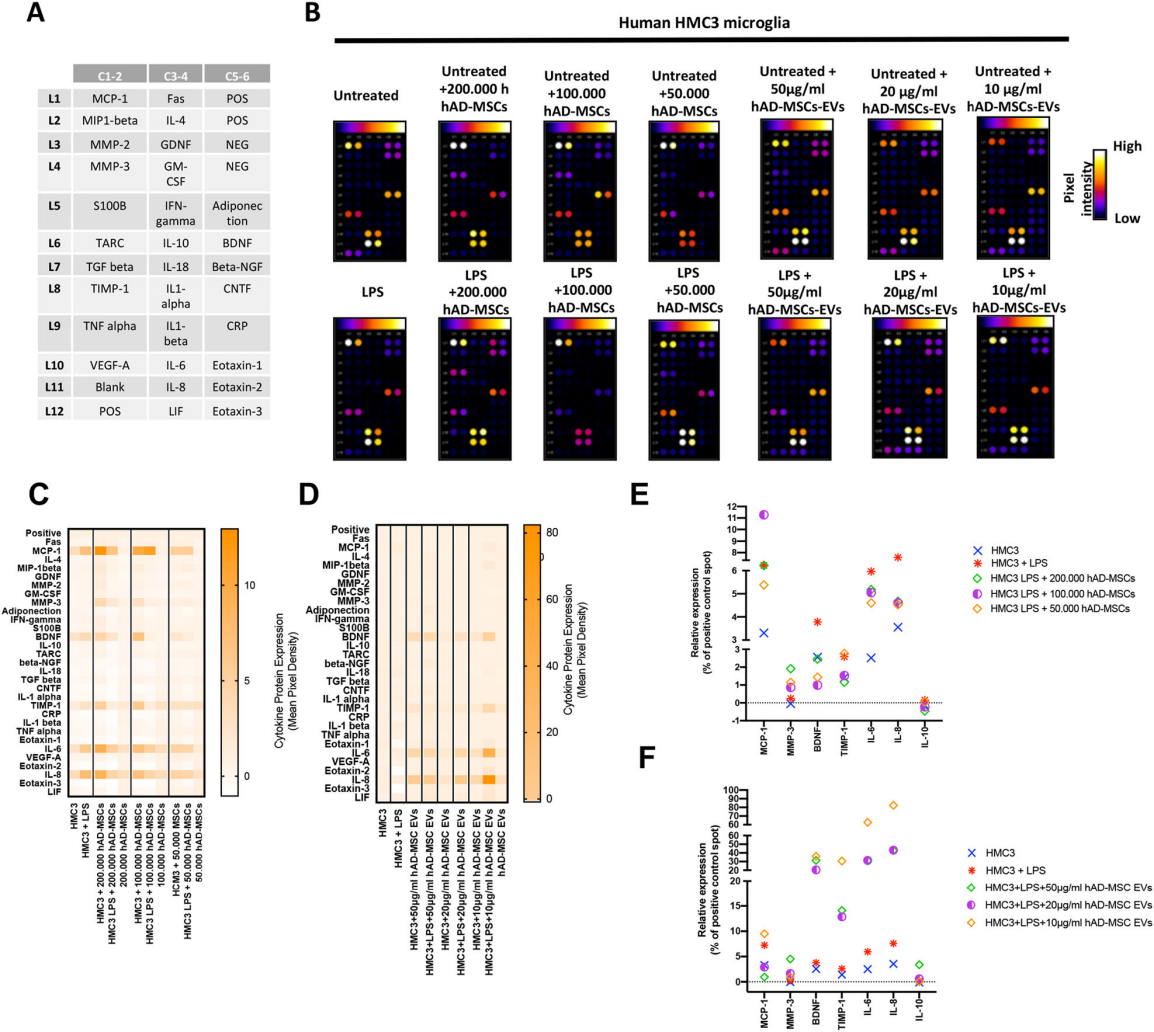


**Fig. 6 Changes in cytokine production in untreated or LPS-stimulated HMC3 microglia mediated by hAD-MSCs and hAD-MSC EVs. A** Gene map of cytokine array. **B** Cytokine arrays, in which human HMC3 microglia have been cultured with human adipose tissue-derived stem cells or extracellular vesicles; all spots are in duplicate. **C, D** Heat map showing relative cytokine expression. **E, F** Relative expression of select cytokines; values are shown as the relative mean pixel density of the duplicate spots.

**Text Corrections:**

**Old Text:** Following LPS activation, the amount of inducible nitric oxide synthase (iNOS) in HMC3 cells significantly increased from 1.49 to 98.51 arbitrary units (Fig. 4B, C). On the contrary, in the presence of hAD-MSCs and hAD-MSC EVs, at all ratios, the level of iNOS was significantly decreased (Fig. 4C).

**New Text:** Following LPS activation, inducible nitric oxide synthase (iNOS) increased in HMC3 cells (Fig. 4B, C). On the contrary, in the presence of hAD-MSCs and hAD-MSC EVs, the level of iNOS was decreased (Fig. 4C).

**Old Text:** Following LPS activation of HMC3 cells, there was a fourfold increase in IL-6 and fivefold increase in IL-8 when compared to resting untreated microglia (Fig. 6C–F).

**New Text:** Following LPS activation of HMC3 cells, there were increases in IL-6 and IL-8 when compared to resting untreated microglia (Fig. 6C–F).

The original article has been corrected

