# Supplementary material for: Human adipose tissue-derived mesenchymal stem cells and their extracellular vesicles modulate lipopolysaccharide activated human microglia
Source: Cell Death Discov. 2021 May 10;7:98. doi: 10.1038/s41420-021-00471-7 (PMC8110535; doi:10.1038/s41420-021-00471-7)
Supplement: Supplementary file 1 — Table, figures and supplementary figures legends [file 41420_2021_471_MOESM1_ESM.docx]

**Table Legends**

**Table 1:** Antibody list

**Figure Legends**

**Figure 1**: Effects of LPS on HMC3 microglia. (A) Representative phase-contrast images show morphological changes in HMC3 microglia after 24h incubation with different doses of LPS. Scale bar, 500 μm (B) Immunofluorescence staining for CD11b (green fluorescent signal), phalloidin (white) and nuclear DAPI staining (blue) was performed. Scale bar, 49 μm. (C) Cell viability after 24h incubation with different doses of LPS was determined by the XTT assay. Values for each treatment are expressed as percentages of the corresponding control (cells untreated). The results shown are the mean ± SEM of triplicates. ** Indicates a significant difference from the LPS-activated group vs control (p < 0.01). (D) LPS-induced ROS generation in HMC3 microglia cells in response to 24 h LPS exposure was determine using celigo imaging cytometer. Representative images are shown. (E) Heat map showing relative cytokines expression. Collected supernatants were subject to cytokine array blots containing 30 different human neurologically relevant proteins. (F) Representation of relative cytokines expression. The values are shown as mean pixel density.

**Figure 2**: Human Adipose tissue-derived Stem Cells and Extracellular Vesicles Characterization. (A) Morphology of isolated hAD-MSCs after 24 hours in culture. Representative images are shown. (B) Western blotting was used to confirm the abundances of extracellular vesicle marker CD63 in isolated extracellular vesicles (EVs), cell culture supernatants (CM) and cell lysates. Ponceau red staining is representative of total protein content. (C) Gene map of cytokine array containing 30 different human neurologically relevant proteins; all spots are in duplicate and (D) Cytokine array membranes. (E) Heat map showing relative cytokines expression. The values are shown as the mean pixel density.

**Figure 3**: Co-culture of human HMC3 microglia cells and human adipose tissue-derived mesenchymal stem cells OR extracellular vesicles. (A) Schematic overview of the co-culture protocol. (B) Fluorescence images of culture HMC3 microglia cells (green) and human adipose tissue-derived mesenchymal stem cells (red) in the presence or absence of 1µg/ml LPS. (C) Fluorescence images of culture HMC3 microglia cells (green) and human adipose tissue-derived mesenchymal stem cell extracellular vesicles (red) in the presence or absence of 1µg/ml LPS. Scale bar 500 µm.

**Figure 4**: Effects of hAD-MSCs on microglia immunoinflammatory phenotype. (A) Immunofluorescence staining for M1 marker CD11b (green), F-actin phalloidin (white) and DAPI (blue). hAD-MSCs are labeled with CellBrite Orange (red) (B) Proteins expression levels of inducible nitric oxide (iNOS). (C) Western blot quantification. Abbreviations: iNOS = inducible nitric oxide.

**Figure 5**: Human adipose tissue-derived stem cells secreted extracellular vesicles enhanced phagocytosis in human HMC3 microglia. Representative images of untreated or LPS-stimulated HMC3 microglia capacity of phagocytize fluorescent-labeled latex beads.

**Figure 6**: Changes on cytokine production in untreated or LPS-stimulated HMC3 microglia mediated by human adipose tissue-mesenchymal stem cells and extracellular vesicles. (A) Gene map of cytokine array; all spots are in duplicate, (B) Image of co-culture cytokine arrays, and human adipose tissue-derived stem cells or extracellular vesicles, and (C-D) Heat map showing relative cytokines expression. (E-F) Representation of cytokines relative expression. The values are shown as the relative mean pixel density.

**Supplementary figures Legends**

**Supplementary figure 1**: Cytokine array changes on MCP-1, IL-6 and IL-8 proinflammatory cytokines production in HMC3 activated microglia mediated by human adipose tissue-mesenchymal stem cells and extracellular vesicles. Collected supernatants were subject to cytokine array blots containing 30 different human neurologically relevant proteins. The values are shown as the mean pixel density.

**Supplementary figure 2**: Changes on TIMP-1 and IL-10 antinociceptive factors and BDNF in HMC3 activated microglia mediated by human adipose tissue-mesenchymal stem cells and extracellular vesicles. Collected supernatants were subject to cytokine array blots containing 30 different human neurologically relevant proteins. The values are shown as the mean pixel density.
