# Supplementary figures and images for: Human adipose tissue-derived mesenchymal stem cells and their extracellular vesicles modulate lipopolysaccharide activated human microglia
Source: Cell Death Discov. 2021 May 10;7:98. doi: 10.1038/s41420-021-00471-7 (PMC8110535; doi:10.1038/s41420-021-00471-7)

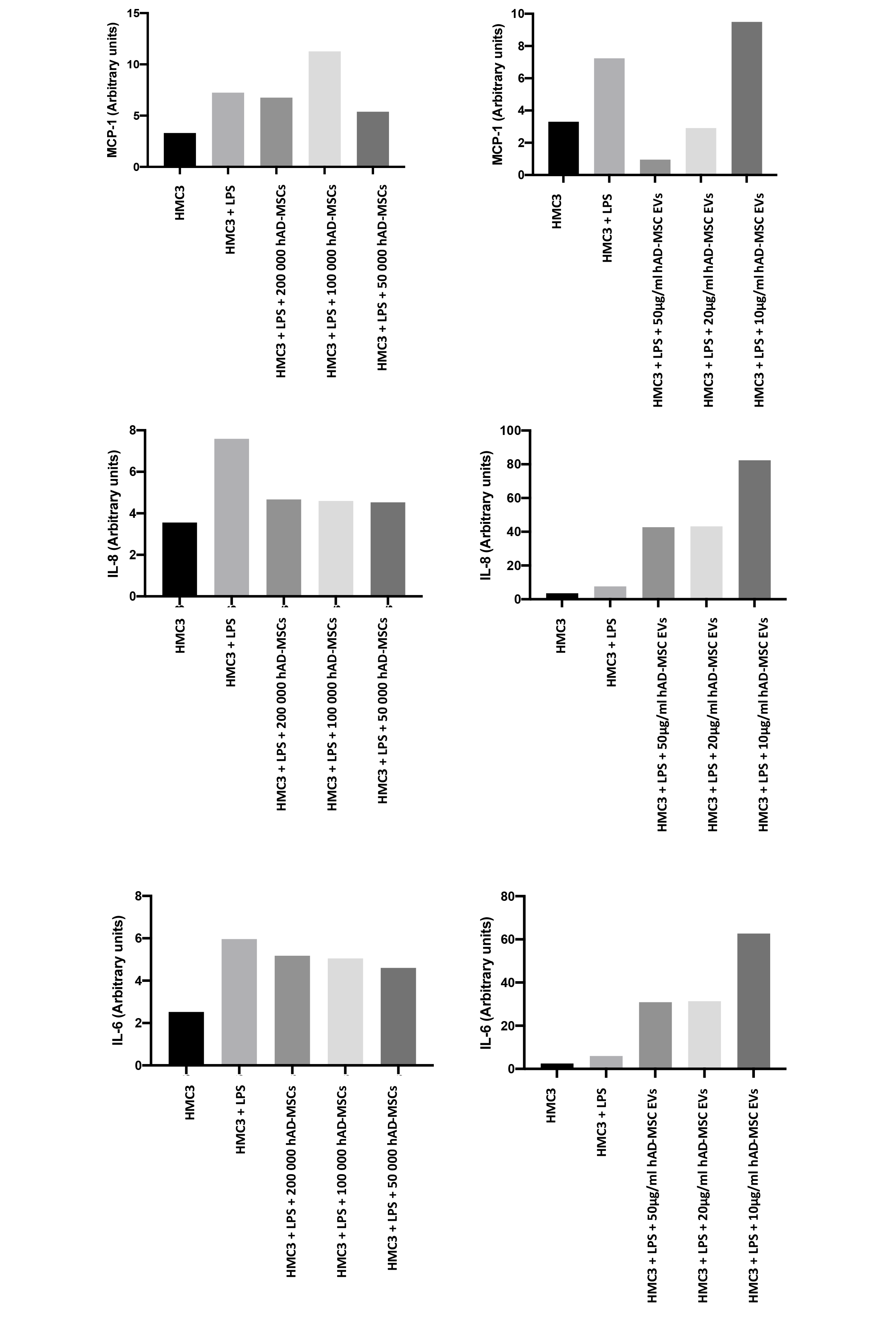

Supplement: Supplementary file 2 — Supplementary Figure 1 [file 41420_2021_471_MOESM2_ESM.tif]

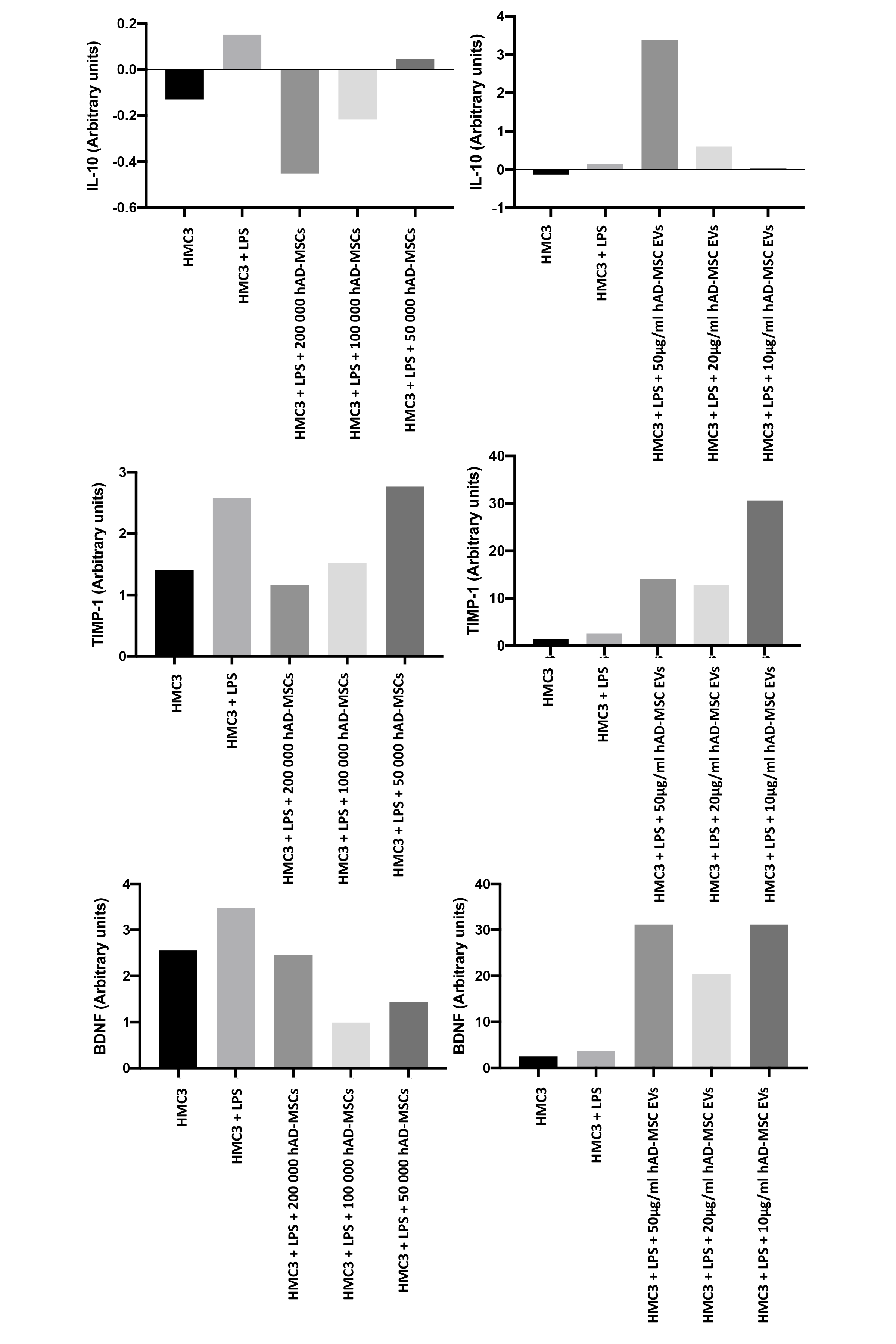

Supplement: Supplementary file 3 — Supplementary Figure 2 [file 41420_2021_471_MOESM3_ESM.tif]
